# Improving the operational forecasts of outdoor Universal Thermal Climate Index with post-processing

**DOI:** 10.1007/s00484-024-02640-6

**Published:** 2024-03-05

**Authors:** Danijela Kuzmanović, Jana Banko, Gregor Skok

**Affiliations:** 1 Faculty of Mathematics and Physics, University of Ljubljana, Jadranska Cesta 19, Ljubljana, 1000 Slovenia; 2 Slovenian Environment Agency, Vojkova 1b, Ljubljana, 1000 Slovenia

**Keywords:** UTCI forecasting, Thermal comfort, Verification, Post-processing

## Abstract

The Universal Thermal Climate Index (UTCI) is a thermal comfort index that describes how the human body experiences ambient conditions. It has units of temperature and considers physiological aspects of the human body. It takes into account the effect of air temperature, humidity, wind, radiation, and clothes. It is increasingly used in many countries as a measure of thermal comfort for outdoor conditions, and its value is calculated as part of the operational meteorological forecast. At the same time, forecasts of outdoor UTCI tend to have a relatively large error caused by the error of meteorological forecasts. In Slovenia, there is a relatively dense network of meteorological stations. Crucially, at these stations, global solar radiation measurements are performed continuously, which makes estimating the actual value of the UTCI more accurate compared to the situation where no radiation measurements are available. We used seven years of measurements in hourly resolution from 42 stations to first verify the operational UTCI forecast for the first forecast day and, secondly, to try to improve the forecast via post-processing. We used two machine-learning methods, linear regression, and neural networks. Both methods have successfully reduced the error in the operational UTCI forecasts. Both methods reduced the daily mean error from about 2.6$^{\circ }$C to almost zero, while the daily mean absolute error decreased from 5$^{\circ }$C to 3$^{\circ }$C for the neural network and 3.5$^{\circ }$C for linear regression. Both methods, especially the neural network, also substantially reduced the dependence of the error on the time of the day.

**Supplementary Information:**

The online version contains supplementary material available at 10.1007/s00484-024-02640-6.

## Introduction

The Universal Thermal Climate Index (UTCI) is a thermal comfort index that quantitatively describes how the human body experiences ambient conditions. It is based on an advanced thermo-physiological model (Pappenberger et al. 2015). It has units of temperature, and it takes into account the effect of air temperature, humidity, wind, radiation, and clothes (Bröde et al. 2012). One of its favorable properties is that it can be used to express thermal comfort throughout the entire range of thermal conditions (i.e., for hot and cold conditions, Zare et al. 2018).

Following the concept of an equivalent temperature, UTCI for a given combination of wind speed, radiation, humidity, and air temperature was defined as the air temperature of the reference environment, which, according to the thermo-physiological model, produces an equivalent dynamic physiological response (Bröde et al. 2012). The reference conditions are 10 m wind of 0.5 m/s, relative humidity of 50%, mean radiant temperature equal to the air temperature, and a person walking at a speed of 4 km/h (Bröde et al. 2012). It is based on a relatively complex Fiala multi-node model of human heat transfer and temperature regulation that can reproduce the human thermal response to a wide range of external climatic conditions (Fiala et al. 2012).

Due to its strengths as a bioclimatic index, the UTCI has been widely used in many studies in bioclimatology and in many related scientific disciplines (Błażejczyk and Kuchcik 2021). For example, assessment of regional and local bioclimate characteristics (Wu et al. 2010; Błażejczyk and Matzarakis 2007; Kingma et al. 2021; Folkerts et al. 2021; Eggeling et al. 2022), urban bioclimate (Czarnecka et al. 2011; Bröde et al. 2013; Nowosad and Wereski 2013; Błażejczyk et al. 2014; Lukić and Milovanović 2020), recreation, tourism and sports (Liu et al. 2022; Krüger 2017; Lindner-Cendrowska and Błażejczyk 2018), epidemiology and health research (Nastos and Matzarakis 2011; Morabito et al. 2014; Urban and Kyselý 2014; Krzyżewska et al. 2017; Romaszko et al. 2017; Błażejczyk et al. 2018; Di Napoli et al. 2018; Skutecki et al. 2019; Urban et al 2021), *UTCI* mapping (Vinogradova 2019; Kuchcik et al. 2021; Milewski 2013; Di Napoli et al. 2018), assessment/forecast of bioclimatic changes (Rozbicki and Rozbicka 2018; Kuchcik et al. 2021; Głogowski et al. 2020; Brecht et al. 2020; Błażejczyk et al 2013; Emerton et al. 2022).

The UTCI is also increasingly used in many countries as a measure of thermal comfort for outdoor conditions and is calculated as part of the operational meteorological forecast (Di Napoli et al. 2021a). Some examples of such use of the UTCI in Europe are: the Czech Republic (since 2019, Novak 2013; Novák 2021), Italy (since 2007, Maracchi 2017), Poland (since 2010, Bröde et al. 2012; IMGW-PIB 2015), Portugal (since 2010, IPMA 2022), and Slovenia (since 2019, ARSO 2021).

At the same time, forecasts of outdoor UTCI tend to have a relatively large error primarily caused by the error of forecasts of relevant meteorological parameters that are used as input for the UTCI (e.g., Novák 2021; Pappenberger et al. 2015). Our goal was to analyze the errors of the operational outdoor UTCI forecasts for the first day and then try to reduce these errors via post-processing by using two machine-learning approaches. We used a relatively simple method (linear regression) and a more complex non-linear method (neural network). We tried to improve the forecasts for Slovenia, which has a relatively dense network of meteorological stations where the forecasted and observed UTCI values could be compared. We used hourly data for seven years (2013–2018 and 2020) at 42 stations.

## Data and methods

###  Universal thermal climate index

As already mentioned, the UTCI is defined as an equivalent air temperature that would produce the same physiological response under a set of reference conditions (Bröde et al. 2012). The UTCI is a function of several environmental parameters and can be defined as follows:

1 $$\begin{aligned} UTCI(T_{\textrm{a}},T_{\textrm{mrt}},e,v_{\textrm{10m}}) = T_{\textrm{a}} + \text {Offset}(T_{\textrm{a}},T_{\textrm{mrt}},e,v_{\textrm{10m}}), \end{aligned}$$

where $T_{\textrm{a}}$ is air temperature, *e* is water vapour pressure, and $v_{\textrm{10m}}$ is wind speed at 10 m height (Bröde et al. 2012). The function Offset represents the deviation of the UTCI from the actual air temperature. Lastly, the mean radiant temperature ($T_{\textrm{mrt}}$) is a measure of the total radiation from the atmosphere and the ground incident on an object from all directions, but instead of expressing this measure as a flux density, it is converted into a temperature via the Stefan-Boltzmann equation (Di Napoli et al. 2020).

The UTCI is based on the Fiala multi-node model of human thermoregulation  (Fiala et al. 2012). However, calculating the UTCI by running the complete Fiala model is computationally expensive, and simpler calculation procedures, such as look-up tables (such as the table presented by Bröde et al. 2012) or polynomial regression methods, are usually used. For example, an approximation of the Offset function by a 6th-degree polynomial that depends on $T_{\textrm{a}}$, $v_{\textrm{10m}}$, *e*, and $T_{\textrm{mrt}}-T_{\textrm{a}}$, can be used (such an example, with 210 polynomial coefficients, was presented by Bröde et al. 2012). Such approximation is used by the BioKlima model and the ALADIN numerical weather prediction model.

### Data description

#### Measurements at meteorological stations

**Fig. 1:**
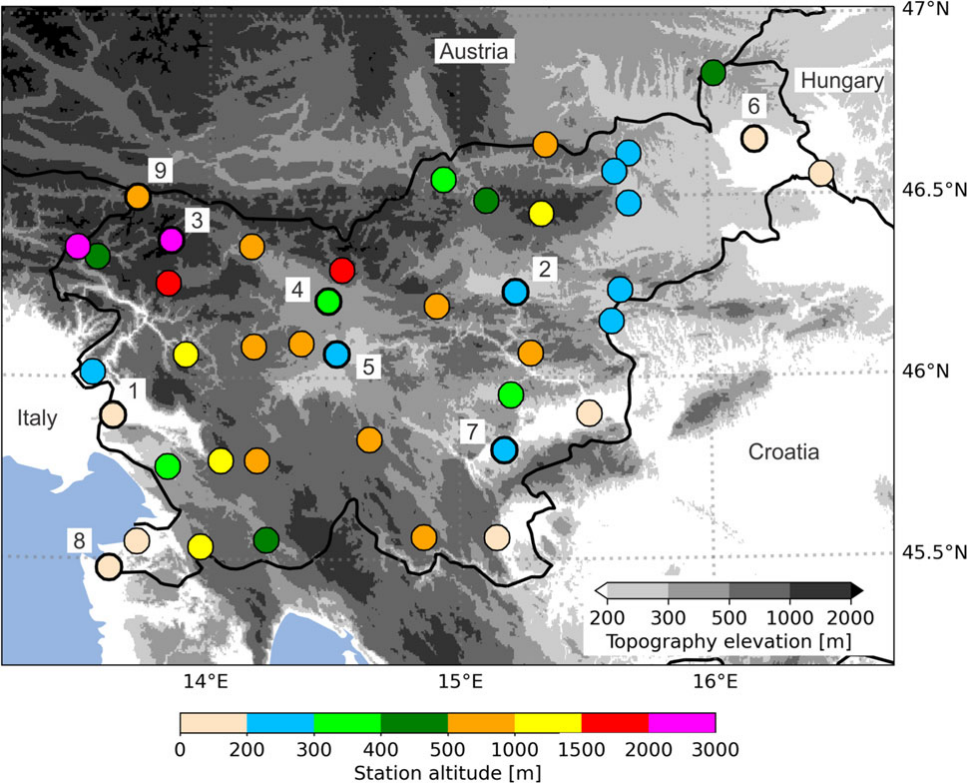
The geographical distribution of meteorological stations used in the study. The circular markers are color-coded to reflect the altitude of each station, with altitudes indicated by the color bar below the figure. The nine stations that were individually analyzed are marked with bold circles and id numbers: 1. Bilje, 2. Celje-Medlog, 3. Kredarica, 4. Letalisce Jožeta Pučnika Ljubljana, 5. Ljubljana-Bežigrad, 6. Murska Sobota-Rakičan, 7. Novo Mesto, 8. Portorož-Letalisce, 9. Rateče. The gray shading represents the topography elevation from the ETOPO Global Relief Model (NOAA National Centers for Environmental Information 2022)

Slovenia is situated at the intersection of four major European geographic regions (the Alps, the Dinarides, the Pannonian Plain, and the Mediterranean). It has very complex terrain with altitudes ranging from 0 to about 3000 m as well as multiple climate types (Kozjek et al. 2017). The meteorological data used in this study was collected on 42 automatic stations, which provide data on an hourly resolution. These stations are part of the Slovenian Environment Agency (SEA) measurement network, and their spatial distribution can be seen in Fig. 1. A list of all stations with some information is shown in Table S6 in the Supplementary Materials. Seven years of data (2013–2018 and 2020 - this period was determined by the availability of the ALADIN model data) from these stations were used in the study.

The SEA employs an automated quality control algorithm that checks for probable errors or inconsistencies in the measurements. For example, it flags values where unusually large changes occurred in a short time or values that are substantially different from values at nearby stations. The values flagged by the automated algorithm are then manually checked by a quality control expert who decides if a particular measurement should be kept in the archive or discarded. The missing values were not used in the analysis (no gap filling or interpolation was performed if a measurement was missing).

Since the stations span a large range of altitudes and multiple climate types, they represent a good dataset for evaluating the performance of the UTCI forecast as well as their post-processing. Crucially, measurements of global solar radiation are also performed at these stations. Having radiation measurements makes estimating the actual value of UTCI more reliable since $T_{\textrm{mrt}}$ can be determined with more accuracy compared to the situation when no radiation measurements are available (in this case, other less accurate proxy parameters, need to be used).

For example, Novák (2021) compared the ALADIN-based UTCI forecasts in the Czech Republic to data from a single automatic weather station for a period of eleven months. Since no radiation measurements were available at the station, they used two proxies for $T_{\textrm{mrt}}$ – they assumed that $T_{\textrm{mrt}}$ is equal either to the air temperatures measured at 2 m or to the temperature measured at 5 cm above the ground. Another example is the study by Di Napoli et al. (2021b) where the authors tried to verify the $T_{\textrm{mrt}}$ and UTCI values derived from ECMWF ERA5-HEAT reanalysis by comparing them to the values they obtained from 177 meteorological stations distributed around the world. Since measurements of radiation were not available at these stations, they had to rely on other parameters, such as air temperature and total cloud cover, to estimate the values of $T_{\textrm{mrt}}$ and UTCI.

In addition to analyzing the combined data from all 42 stations, we also selected nine stations (marked with numbers in Fig. 1), representing distinct geographical and climatic characteristics in Slovenia, to analyze how the UTCI forecast post-processing performs if done separately for each station (as opposed to all the stations together).

From the centrally located Ljubljana Basin, which exhibits a Subcontinental climate (Kozjek et al. 2017), we selected the Ljubljana-Bežigrad station in the capital city and the Jože Pučnik Airport Ljubljana station to represent both urban and countryside locations. From the NW part of Slovenia, we selected the Kredarica station, which has the highest altitude and an Alpine climate, and the Rateče station, located at the bottom of an alpine valley at a much lower altitude and exhibiting a Subalpine climate (Kozjek et al. 2017). From the SW part, which exhibits a Submediterranean climate (Kozjek et al. 2017), we selected the Portorož-Letališče station at the coast and the Bilje station located more inland. From the Celje Basin, we selected the Celje-Medlog station, while from the SE part of Slovenia, we selected the Novo Mesto station, with both stations exhibiting a Subcontinental climate (Kozjek et al. 2017). From the NE part of Slovenia, which also exhibits a Subcontinental climate (Kozjek et al. 2017), we selected the Murska Sobota-Rakičan station.

The software BioKlima 2.6 (Błażejczyk 2017) was used to calculate the UTCI values from the meteorological measurements. BioKlima is a user-friendly software for the Windows operating system that offers various methods for bioclimatic studies and can calculate around 60 different biometeorological and thermophysiological indices. It has been used successfully in previous studies for UTCI calculations (Liu et al. 2022; Rozbicki and Rozbicka 2018; Zare et al. 2018).

As $T_{\textrm{mrt}}$ is not directly measured in Slovenia, BioKlima calculated it based on other parameters such as sun altitude and measurements of global solar radiation and temperature (Wu et al. 2010; Błażejczyk and Matzarakis 2007; Błażejczyk 2005). The Sun’s altitude can be directly inputted or calculated by BioKlima from parameters such as month, day, hour, minutes, and latitude.

The input data for UTCI calculations in the BioKlima model consisted of month, day, hour, minutes, latitude, and measurements of air temperature, relative humidity, wind speed at 10 m height, and global solar radiation.

#### ALADIN model forecasts

The ALADIN model (Aire Limitée Adaptation Dynamique Développement International, Termonia et al. 2018) is a numerical weather prediction model that has been used operationally in Slovenia since 1997 by SEA. The model configuration includes a horizontal resolution of 4.4 km, 87 terrain-following sigma vertical levels, a domain with $421 \times 421$ grid points, and a time step of 180 s (Wang et al. 2018). The extent of the model domain is shown in Fig. S4 in the Supplementary Materials.

The UTCI calculation has recently been incorporated into the ALADIN using the 6th-degree polynomial approximation mentioned in “Universal thermal climate index”. The forecasted value of UTCI is calculated in the ALADIN in a post-processing manner with the forecasted values of some of the model’s basic variables used as input. The mean radiant temperature ($T_{\textrm{mrt}}$) is a key component in the calculation of UTCI, and in the ALADIN model, it is obtained by taking into account various shortwave and longwave fluxes (for details on how the $T_{\textrm{mrt}}$ is calculated in ALADIN please refer to Weihs et al. 2011).

The SEA provided the data for the ALADIN-based forecasts. Seven years (2013–2018, and 2020) of archived operational model outputs were used for the analysis (the forecast data for 2019 was not readily available in the SEA archive, and providing it would demand a significant investment of both time and resources). The data was provided on a spatial resolution of 4.4 km and a temporal resolution of one hour. The data were provided for the first 24 hours of the forecasts, initialized at 00 UTC. The ALADIN forecast is integrated for 72 hours, but only the outputs for the first 24 hours are archived by SEA and could thus be used in the analysis. The model data from the grid point that was nearest to the location of the station was used for the analysis.

### Methods

#### Verification metrics

The mean error (ME) and mean absolute error (MAE) were used as criteria to evaluate the model performance. ME is the difference between the average forecast and average observation and expresses the bias of the forecasts (Wilks 2006). It is defined as

2 $$\begin{aligned} ME=\frac{1}{N} \sum _{i=0} ^{n} \left( f_{i} - o_{i}\right) \>, \end{aligned}$$

where $f_i$ and $o_i$ and the forecasted and observed values, respectively, and *N* the number of data. ME closer to zero indicates a better forecast (von Storch and Zwiers 1999).

The MAE is the arithmetic average of the absolute values of the differences between the pairs of forecasted and observed values and can be interpreted as a typical magnitude for the forecast error in a given verification data set (Wilks 2006). It is defined as

3 $$\begin{aligned} MAE= \frac{1}{N}{\sum _{i=1}^n |f_i - o_i |}\>, \end{aligned}$$

A lower MAE indicates a better forecast (von Storch and Zwiers 1999).

#### Neural network

Modern machine learning techniques, especially Neural Networks (NNs), are increasingly used to improve specific aspects of weather prediction (e.g. Reichstein et al. 2019; Palmer 2020; Schultz et al. 2021). Due to their strengths and successful use by researchers in many previous studies, we decided to use the NN to try to improve the accuracy of the ALADIN-based UTCI forecasts.

We used various parameters outputted by ALADIN (e.g., forecasted UTCI, air temperature, humidity, wind speed, cloudiness), as well as some other parameters (e.g., station altitude) as input variables for the NN. The output from the NN was a corrected value of UTCI forecast. To train the NN, we also used the UTCI values derived from the measurements at the stations.

To prepare the data for training the NN and using it for Linear Regression (LR, Goodfellow et al. 2016), we normalized the input and output variables to a range between 0 and 1 using the MinMaxScaler module from the Python scikit-learn library (Pedregosa et al. 2011). This normalization process ensures that all variables have a similar scale, which helps with the training of NN. Next, we randomly shuffled the data to remove any temporal sequences and divided it into training (90%) and test (10%) sets. The training set was used to train the NN (and LR), while the test set was used to evaluate its performance once the training had finished.

We used fully-connected feedforward NNs (Goodfellow et al. 2016) that consisted of several layers of neurons that are fully connected – namely, all the neurons in one layer are connected with all the neurons in the previous layer. The values of neurons in each layer were thus determined from all the values in the previous layer, while the input parameters determined the values of the neurons in the input layer. We implemented the NNs in Python with the TensorFlow library (Abadi et al. 2016)

**Fig. 2:**
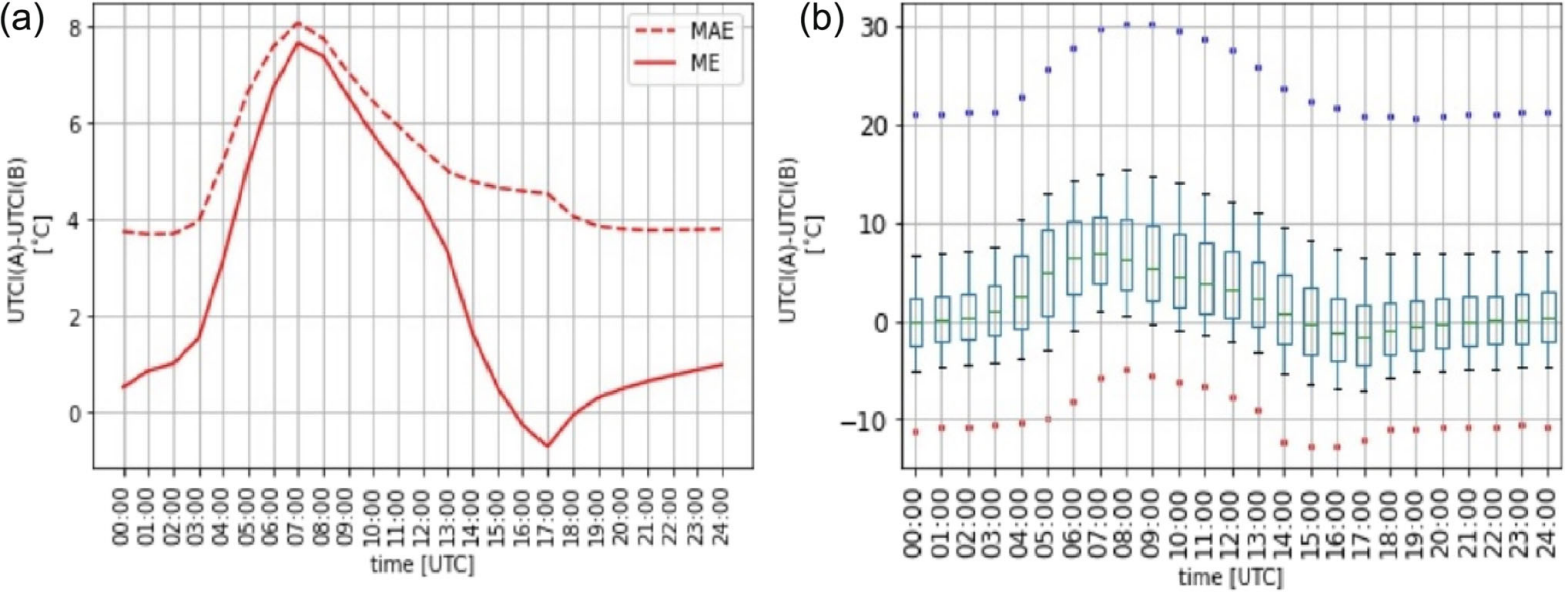
Verification of ALADIN-based UTCI forecasts for all stations in the seven years that were analyzed (2013–2018 and 2020). (a) ME and MAE for each hour of the day. (b) The distribution of differences between the forecasted and observed UTCI values. The median is represented by the green line within the box, which represents the values between the 25$^{\textrm{th}}$ and 75$^{\textrm{th}}$ percentiles. The whiskers show the range from the 10$^{\textrm{th}}$ to 25$^{\textrm{th}}$ percentile and from the 75$^{\textrm{th}}$ to 90$^{\textrm{th}}$ percentile. The red and blue dots represent the 1$^{\textrm{st}}$ and 99$^{\textrm{th}}$ percentile, respectively

The architecture of the NN consisted of an input layer, three hidden layers, and an output layer. The number of neurons in the input layer varied based on the number of input parameters, while the hidden layers had 5, 3, and 1 neurons, respectively. The output layer had only one neuron, whose value determined the forecasted UTCI value. We also experimented with more complex NN architectures consisting of many more neurons arranged in more layers but found that more complex NNs did not perform substantially better. We used the LeakyReLU activation function (Maas 2013) for the hidden layers and a linear activation function for the output layer. The Adam optimizer (Kingma and Ba 2014) and the MAE loss function were employed during training. The NN was trained for 100 epochs (testing showed that using more epochs did not improve the results).

We evaluated the performance of the NN and LR by calculating the ME and MAE for the test set. We trained the NN and LR using data from all the stations together (in this case, we used a batch size of 1000 to speed up the training process). Additionally, we trained the NN and LR separately for the nine selected stations to assess their performance on a per-station basis with a batch size of 100.

To evaluate the performance of various approaches, we compared the forecasted UTCI values with the observed UTCI values. This comparison was made for the independent test set (which was not used for training the NN or LR) and was made separately for the NN-based forecasts, the LR-based forecasts, and the non-post-processed ALADIN-based forecasts of UTCI.

**Fig. 3:**
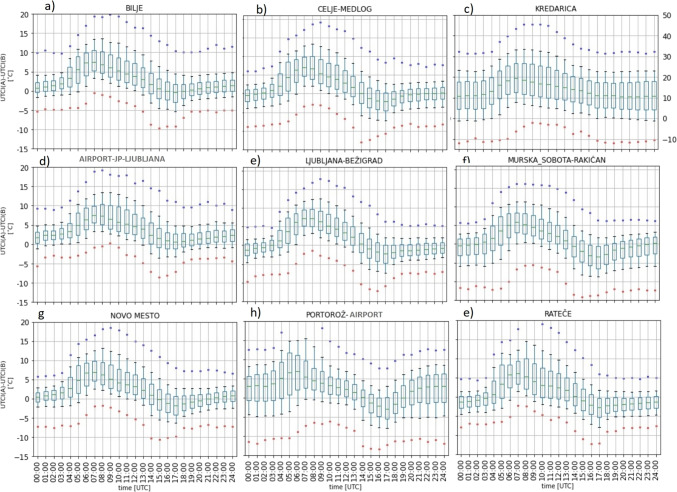
Same as Fig. 2b, but for selected nine stations shown in bold font in Fig. 1. The y-axis of each subfigure has the same range for all stations from -15$\,^{\circ }$C to 20$\,^{\circ }$C, except for the Kredarica station, which has a range from -10$\,^{\circ }$C to 50$\,^{\circ }$C

#### Linear regression

NNs represent a relatively advanced and complex machine-learning approach. We also wanted to assess whether employing such a complex approach yields substantial advantages over a simpler approach. This is why we also used Linear Regression (LR), which is one of the simplest methods that can be used to explain a relationship between a chosen dependent variable and one or more explanatory variables. In LR, the dependent variable can be expressed as a linear combination of explanatory variables as

4 $$\begin{aligned} Y= b_0 + b_{1} X_{1} + b_{2} X_{2} + ... + b_{k} X_{k}, \end{aligned}$$

where *Y* is the dependent variable while $X_1,...,X_k$ are the explanatory variables (Darlington and Hayes 2017). In our case, the explanatory variables are the same normalized input variables that were also used with the NNs. $b_{0}$ is the regression constant, and $b_{1},...,b_{k}$ are regression coefficients (Darlington and Hayes 2017) that are determined using the least squares method that minimizes the sum of the squared differences between the expected and predicted outputs (Darlington and Hayes 2017).

The sign and size of a regression coefficient determine how a particular explanatory variable influences the dependent variable. For example, if the sign of the coefficient is positive, the dependent variable value will increase when the explanatory variable value increases, and vice versa. Moreover, if the coefficient’s absolute value is larger, the influence on the dependent variable will also be larger, which can be used to determine which explanatory variables will tend to have a larger influence on the dependent variable.

We used the Python scikit-learn library (Pedregosa et al. 2011) to implement the LR, and we used the same input/output variables as with the NN.

## Results

### Verification of ALADIN-based UTCI forecasts

The first goal of this study was to verify operational ALADIN-based UTCI forecasts for Slovenia. Figure 2a shows the ME and MAE with respect to the time of the day. The solid line represents ME, while the dashed line represents MAE. The daily average ME of the UTCI forecasts is 2.56$\,^{\circ }$C, but its value changes a lot with respect to the time of the day. For example, at 7 am, the ME is almost 8$\,^{\circ }$C while it is close to zero in the evening. The average MAE is 5.02$\,^{\circ }$C. MAE also has a peak at 7 am, exceeding 8$\,^{\circ }$C, while it is about 4$\,^{\circ }$C in the evening. Figure 2b shows the distribution of differences between the forecasted and observed UTCI values. We can note that the median follows the ME course, with maximal value 6.9$\,^{\circ }$C reached at 07:00 and minimal value -1.5$\,^{\circ }$C obtained at 17:00. In addition, we can observe that the interquartile range is larger during the daytime, with a maximum value of 8.4$\,^{\circ }$C reached at 5:00 and a minimum value of 4$\,^{\circ }$C obtained at midnight. The rather large distance between the $1^{\textrm{st}}$ and $99^{\textrm{th}}$ percentile (red and blue dots) of about 35$\,^{\circ }$C indicates issues with ALADIN forecasts. For example, in about 2% of cases, ALADIN overestimates the UTCI value for more than 20$\,^{\circ }$C, while in about 1% of cases, ALADIN underestimates the UTCI value for more than 10$\,^{\circ }$C.

The results in Fig. 2 can be compared to those from Novák (2021) who tried verifying the ALADIN-based UTCI forecasts in the Czech Republic. It is interesting to note that they got quite different results. For example, they found that the ALADIN model tended to underestimate the UTCI values and that the largest error was during the night, while the error was smaller during the day and the smallest in the mornings and afternoons. It should also be stressed that the ALADIN model used in Slovenia (by SEA) is not identical to the ALADIN model used in the Czech Republic (by the Czech Hydrometeorological Institute) as the different branches of the original ALADIN model have been developed separately for quite a long time. Nevertheless, it is important to highlight the differences between the two studies. Novák (2021) used eleven months of data from a single station, while we used seven years of data from 42 stations. They also did not have any radiation measurements available at their station and used temperatures measured at heights of 2 m and 5 cm as proxies for $T_{\textrm{mrt}}$, while on our stations, measurements of global solar radiation were available.

Figure 3 shows the results for the nine selected stations. Kredarica stands out with the highest median with a maximal median of 18.57$\,^{\circ }$C at 7:00 and a wider spread, where the maximal interquartile range was 15,85$\,^{\circ }$C at 10:00. In overall, we can note that its mean errors tend to be about 10$\,^{\circ }$C larger than at other stations. Kredarica is a high-altitude station located at an altitude of about 2500 m. The errors are likely caused by the difference between the station’s altitude in the model and the actual altitude of the station. Portorož-Airport also shows a wider interquartile range in the first hours, with a maximal value of 10.86$\,^{\circ }$C reached at 5:00. This wide scatter of the errors is possibly influenced by wind forecasts or measurement errors near the sea.

The impact of station altitude on the error is evident in the box plots. To verify this, we categorized stations into two groups based on altitude: those below or equal to 509 m and those above. The results are shown in Fig. S1 in the Supplementary Materials. For both groups, the ME and MAE errors display a similar dependence on the time of the day (with the largest errors in the early morning), but the errors for the low altitude group tend to be 3 to 6$\,^{\circ }$C lower than the errors of the high altitude group.

An investigation of several cases with exceptionally large errors (two such examples are shown in the Supplementary Materials in Section S2) identified several possible causes for these differences:

- Incorrectly forecasted cloud cover, humidity, and surface radiation balance: These factors can have an impact on the calculation of $T_{\textrm{mrt}}$, particularly during the morning hours when the maximum error was observed. Based on the analysis we performed, it was observed that errors in $T_{\textrm{mrt}}$, and consequently in UTCI, tended to be the largest during periods when there were rapid changes in these values, such as during the transition from day to night.
- Incorrectly forecasted wind speed or temperature: Inaccurate forecasts of wind speed or temperature can also contribute to errors in the UTCI prediction.
- Large difference between the station’s altitude in the model and the actual altitude of the station (the station altitudes are shown in Table S6 in the Supplementary Materials). This disparity in altitude can substantially impact the accuracy of radiation forecasts, as the actual altitude of the stations may deviate from the model’s predictions.
- Specific micro-meteorological conditions at the locations of meteorological stations: Factors such as relief variability at a scale smaller than the spatial resolution can influence the accuracy of forecasts. For instance, stations located within urban heat islands may experience differences between measured meteorological parameters and the forecasted values, unlike stations in rural areas with more natural surroundings and no obstacles (although we did not observe a substantial urban heat islands effect since the station at Ljubljana-Bežigrad, which is situated in an urban environment, had one of the smallest errors). Proximity to hills or the sea can also have a notable effect on the model’s ability to predict the UTCI.
- Possible errors in measurements: It should be noted that the station data from 2014 onward has been quality controlled; however, there is still a possibility of errors being present in the measurements that could impact the verification of the forecasts.
- Approximations in determining solar elevation in the BioKlima model: The method used to determine solar elevation in BioKlima may involve approximations, which could influence the results.
- Lack of detailed explanations of calculations in BioKlima: The lack of comprehensive explanations regarding the calculations in BioKlima may pose challenges in understanding the accuracy and reliability of the verification.

We also investigated the seasonal dependence of errors. The results are shown in Fig. S2 in the Supplementary Materials. All four seasons exhibit the same hourly pattern of errors, with the error being the largest in the early morning hours. However, the exact timing, as well as the amplitude of the error maximum, varies across seasons.

In the summer, the largest ME of 8.34$\,^{\circ }$C was at 5:00, while in the autumn, the largest ME of 8.18 $\,^{\circ }$C was at 7:00. For the winter, we observed the largest ME of 10.3$\,^{\circ }$C at 8:00, which was the largest ME of all seasons. During the spring, the ME peaked with 8.9$\,^{\circ }$C at 6:00. The timing of the error peak is probably linked to the time of sunrise, which comes later in the winter than it does in the summer – this is why the error peak in winter happens a few hours later than in the summer. A similar conclusion can also be reached for the MAE error. Generally, both errors tend to be the largest in winter and the smallest in summer. This result is somewhat surprising as we had anticipated larger errors during the summer when the model might encounter challenges in forecasting cloudiness due to convection. Previous analyses revealed the sensitivity of the UTCI to wind speed values, suggesting that the higher errors during winter may be attributed to differences in forecasted and measured wind speeds. The model might also have problems successfully forecasting a relatively thin radiation fog near the surface, which frequently develops in many regions in Slovenia during the night, especially in winter. Such fog affects how fast the temperature increases in the morning and also affects the amount of radiation that reaches the ground. Many regions in Slovenia are also affected by downslope mountain winds and cold-air pools, which frequently form during the night in complex terrain. Since these phenomena can be small and relatively shallow, they might not be forecasted correctly by the model, which could result in the temperature increasing too fast in the forecast in the morning.

### Post-processing of UTCI forecasts

#### Post-processing the combined data from all the stations

The second goal of this study was to try to improve the ALADIN-based operational UTCI forecasts by using post-processing with LR and NN.

First, we wanted to try to obtain a single NN and LR model that could be used for post-processing on all the stations. Thus, we used the combined data from all 42 stations to train the NN and obtain the optimal LR coefficients.

We first tried to identify the relationship between different meteorological parameters and UTCI values by calculating correlation coefficients (not shown). Based on these findings, we prepared six different setups with different sets of input parameters that were used as predictors by the LR and NN (a detailed description of the setups is provided in Table S1 in the Supplementary Materials). Since the initial values of the weights in the NN are determined randomly, each training realization of the NN can produce a somewhat different NN. This is why we performed the NN training three times for each setup and selected the training realization with the smallest MAE error.

**Fig. 4:**
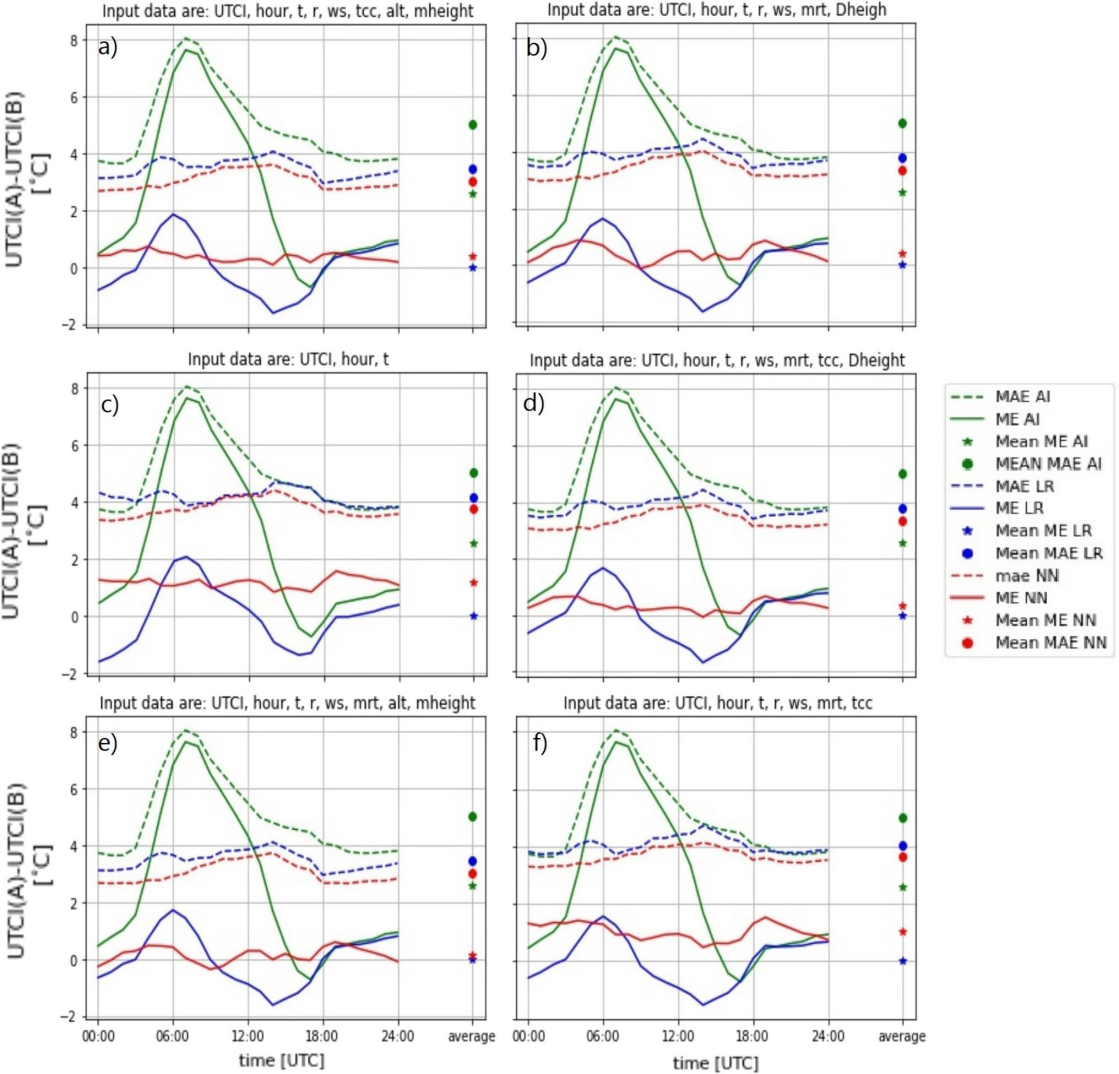
The values of ME and MAE with respect to the hour of the day for all stations for the test set for the six different setups (see Table S1 in the Supplementary Materials for details). From (a) to (f), setups 1, 2, 3, 4, 5, and 6, respectively. The ME is depicted as a solid line and MAE as a dashed line. The results for LR are represented in blue, NN in red, while the results for the uncorrected ALADIN forecasts are shown in green color. The circles on the right side of each graph show the daily average ME and MAE values, which are also shown in Table S2 in the Supplementary Materials

The performance of LR and NN for all six setups is shown in Fig. 4. This visual representation provides insights into the effectiveness of the post-processing methods for improving the accuracy of ALADIN-based UTCI forecasts. Numerical values of daily mean ME and MAE errors for each setup are given in the Supplementary Materials in Table S2.

From the graphs in Fig. 4, it is evident that both methods successfully improve the ALADIN forecast of UTCI in all six setups, resulting in smaller ME and MAE errors. For example, the ME and MAE errors of the uncorrected forecasts for the test set were 2.57$\,^{\circ }$C and 5.02$\,^{\circ }$C, respectively. On the other hand, the ME for the LR was zero for all six setups and the MAE was between 3.47$\,^{\circ }$C and 4.15$\,^{\circ }$C, while the ME for the NN was between 0.15$\,^{\circ }$C and 1.18$\,^{\circ }$C and the MAE between 3.03$\,^{\circ }$C and 3.77$\,^{\circ }$C.

Both methods, especially the NN, also substantially reduce the dependence of the error on the time of the day. For the NN, the forecasts of UTCI in the morning tend to be as accurate as those in the afternoon or night. The same could be said for the MAE error of LR, while its ME does exhibit some hourly dependence with overestimation in the first half of the day and underestimation in the second half.

According to the daily average values of ME and MAE (shown in the Supplementary Materials in Table S2), the NN method outperforms the LR method in terms of MAE, while the LR method tends to have a bit better ME. However, even though the daily average ME for the LR method is almost zero for all setups, LR exhibits a pronounced dependence of ME on the time of the day, which is not present in the case of NN.

The best results for both methods were obtained with Setup 5, which uses the following input parameters: ALADIN UTCI forecast, the hour of the day, forecasted air temperature, forecasted relative humidity, forecasted wind speed, forecasted $T_{\textrm{mrt}}$, station altitude, and the altitude of the station in the model. In this case, the daily average ME and MAE errors for LR were 0.00$\,^{\circ }$C and 3.47$\,^{\circ }$C, respectively, and for NN they were 0.15$\,^{\circ }$C and 3.03$\,^{\circ }$C, while the errors for the uncorrected ALADIN forecasts were 2.57$\,^{\circ }$C and 5.02$\,^{\circ }$C. This means that both post-processing methods reduced the ME from about 2.6$\,^{\circ }$C to almost zero, while the mean absolute error decreased from 5$\,^{\circ }$C to 3$\,^{\circ }$C for the NN and 3.5$\,^{\circ }$C for the LR. Interestingly, including station altitude and model altitude as input parameters yielded better results than using only the difference between these two quantities (this is the only difference between setups 1 and 5).

**Fig. 5:**
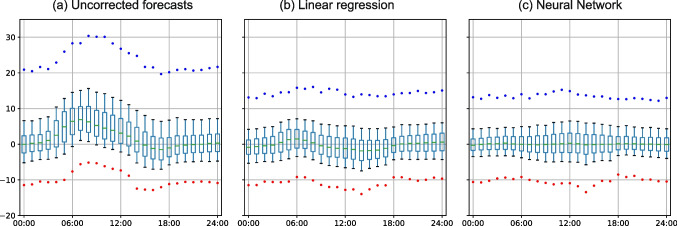
The distribution of differences between the forecasted and observed UTCI values for the data from all the stations evaluated for the test set. Results for Setup 5 are shown. (a) the uncorrected forecasts. (b) the forecasts post-processed by LR. (c) the forecasts post-processed by NN

Analysis of the LR coefficients (shown in the Supplementary Materials in Table S3) revealed that the parameters that have the largest influence on the resulting UTCI value in the LR are the uncorrected ALADIN forecast of UTCI and forecasted air temperature. The coefficients for forecasted $T_{\textrm{mrt}}$ are negative, with values around -0.15. The coefficients for forecasts of cloudiness, relative humidity, wind speed, and the hour of the day are all close to zero. The same is true for the difference between the model and station altitude, while the coefficients for the station and model altitudes tend to be around -0.25 and 0.15, respectively, indicating that the LR uses them.

Figure 5 shows the distribution of differences between the forecasted and observed UTCI values for Setup 5. In addition to moving the median value of the differences closer to zero, both LR and NN also manage to reduce the spread of differences. For example, the mean distance between the 25th and 75th percentile reduces from 6.1$\,^\circ$C to 5.0$\,^\circ$C (LR) and 4.2$\,^\circ$C (NN), the distance between the 10th and 90th percentile reduces from 13.4$\,^\circ$C to 10.5$\,^\circ$C (LR) and 9.2$\,^\circ$C (NN), while the distance between the 1st and 99th percentile reduces from 33.5$\,^\circ$C to 25.3$\,^\circ$C (LR) and 23.8$\,^\circ$C (NN).

**Fig. 6:**
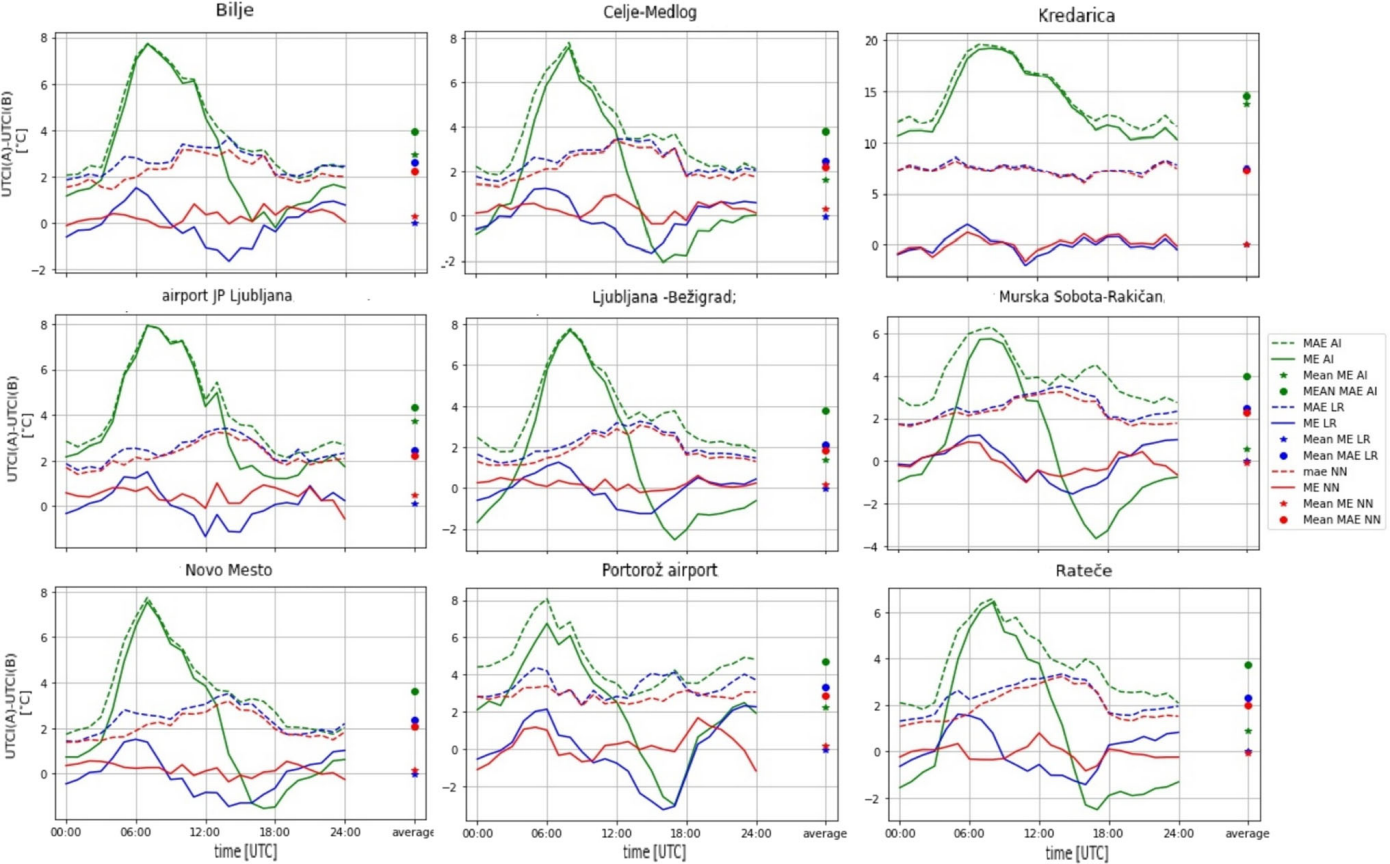
Same as Fig. 4, but for the post-processing done separately on the data from each of the nine selected stations shown with bold font in Fig. 1. Setup 5 was used for all the stations

#### Post-processing the data from individual stations

In “Post-processing the combined data from all the stations”, we tried to obtain a single NN and LR model that would work well for all 42 stations. Here, we tried a different approach by training/optimizing the NN and LR models on data from each of the nine selected stations separately, thereby obtaining a different NN and LR model for each station. The goal was to see how well the post-processing methods perform when optimized and used on data from individual stations.

We used the same input parameters as in Setup 5 (which performed the best with combined data from all the stations) but excluded the station altitude and the station’s altitude in the model since these are constant for a specific station. As before, we trained the NN for each station three times and selected the network with the lowest MAE. The results for all nine stations are depicted in Fig. 6, with the numerical values of daily average ME and MAE errors provided in the Supplementary Materials in Table S4.

From the graphs in Fig. 6, it is evident that both methods yield better results and smaller errors compared to uncorrected ALADIN forecasts for all nine selected stations. For example, for NN the ME reduction was between 0.65$\,^{\circ }$C and 12.68$\,^{\circ }$C, while the MAE reduction was between 1.57$\,^{\circ }$C and 7.27$\,^{\circ }$C. For LR the ME reduction was between 0.57$\,^{\circ }$C and 12.78$\,^{\circ }$C, while the MAE reduction was between 1.26$\,^{\circ }$C and 7.11$\,^{\circ }$C.

Similarly to the analysis done for all 42 stations, NN outperforms the LR in terms of MAE, while LR has a bit better daily average ME (0.00$\,^{\circ }$C vs. 0.16$\,^{\circ }$C, when averaged over the results for all six stations), although both methods have ME close to zero. Interestingly, in terms of MAE, both NN and LR substantially improve the forecasts in the morning hours (when the errors of the uncorrected forecasts are the largest), while during the afternoon and the night, the MAE improves only slightly. Consequently, after the post-processing, the largest MAE error for most stations is in the afternoon and not in the morning, as is the case for uncorrected forecasts.

The station on Kredarica is a special case. As already mentioned, this is a high-altitude station located at an altitude of approximately 2500 m. The errors of the uncorrected forecasts are the largest at this station ($ME = 12.76~^{\circ }$C, $MAE= 14.55~^{\circ }$C), and while both NN and LR substantially improve the forecasts, with ME being close to zero for both methods, the MAE error nevertheless remains large compared to the other stations (i.e., 7.28$\,^{\circ }$C and 7.44$\,^{\circ }$C, for the NN and LR, respectively). Contrary to other stations, the two methods successfully reduce the MAE errors for all hours of the day, and the error does not exhibit dependence on the time of the day.

On the other hand, out of the nine stations, the best results (lowest MAE) are observed for the Ljubljana-Bežigrad station, with MAE reduced from 3.75$\,^{\circ }$C to 1.86$\,^{\circ }$C for NN and 2.10$\,^{\circ }$C for LR.

Similarly to the analysis of the data from all stations, analysis of the size of LR coefficients (shown in the Supplementary Materials in Table S5) revealed that the parameters that have the largest influence on the resulting UTCI value in the LR, are the uncorrected ALADIN forecast of UTCI and the forecasted air temperature. The values of coefficients for other parameters tend to be close to zero.

We also analyzed the distribution of differences between the forecasted and observed UTCI values at three stations: Kredarica, Ljubljana-Bežigrad, and Portorož Airport. The results are shown in the Supplementary Materials in Fig. S3.

For the Kredarica station, which is a high-altitude station with the largest errors, the LR and NN managed to move the median value of the differences closer to zero but were unable to reduce the spread of differences. For example, in the uncorrected forecasts, the mean distances between the 25th-75th, 10th-90th, and 1st-99th percentiles were approximately 14$\,^{\circ }$C, 25$\,^{\circ }$C, and 44$\,^{\circ }$C, respectively, and LR and NN could not reduce these for more than 2$\,^{\circ }$C.

On the other hand, for the Ljubljana-Bežigrad and Portorož Airport stations, the post-processing methods managed to move the median value of the differences closer to zero and reduce the spread of differences. For example, at the Ljubljana-Bežigrad station, mean distances between the 25th-75th, 10th-90th, and 1st-99th percentiles were 4.1$\,^{\circ }$C, 8.2$\,^{\circ }$C, and 16.4$\,^{\circ }$C, respectively. The LR managed to reduce these to 3.2$\,^{\circ }$C, 6.2$\,^{\circ }$C, and 12.9$\,^{\circ }$C, and the NN to 2.7$\,^{\circ }$C, 5.8$\,^{\circ }$C, and 12.6$\,^{\circ }$C.

## Discussion and conclusions

The goals of the study were to analyze errors in the operational forecasts of outdoor UTCI in Slovenia and to improve these forecasts by using post-processing.

The verification of the UTCI forecasts showed that the average ME and MAE errors were typically a few degrees Celsius (about 2.6$\,^{\circ }$C and 5$\,^{\circ }$C, respectively) but depended greatly on the time of the day and were the largest in the morning. There was also a prominent influence of the altitude – namely, the errors were markedly larger at the stations at higher altitudes. Moreover, the errors also exhibited a pronounced seasonal dependency, with the largest errors in winter and the smallest in summer. There were also some situations with very large errors (e.g., $> 20~^\circ$C), which can be caused, for example, by incorrectly forecasted meteorological parameters that strongly influence the UTCI, like cloud cover, temperature, and wind.

Post-processing by NNs or LR substantially improved the accuracy of the UTCI forecasts. Both methods, especially the NNs, also substantially reduced the dependence of the error on the time of the day. Generally, NNs outperformed the LR method, but the difference between the two was not very large, which was surprising since we expected NNs to be substantially better than LR. The NNs we used were relatively simple, and we also tried to use more complex NNs, but they did not perform substantially better. It seems that the simple NNs already managed to correct the most obvious errors that frequently occur in the forecasts, while even the more complex NNs could not help much with the more complicated errors that might occur due to specific weather situations being substantially misdiagnosed in the forecasts.

Overall, the study provides important insights into the typical sizes and properties of errors of operational forecasts of outdoor UTCI and how these forecasts can be post-processed to increase accuracy. Although the study was based on data from a relatively small geographical region, the substantial number of stations used in the analysis, the relatively long analysis period of 7 years, the large climate variability and complex orography of Slovenia make the results more generally relevant. Moreover, the radiation measurements taken at the stations enhance the accuracy of estimating $T_{\textrm{mrt}}$, thereby making the estimation of the true UTCI and the forecast error more reliable. The two presented post-processing approaches show clear benefits by substantially increasing the accuracy of the UTCI forecasts and could also be used in other parts of the world. Their use could improve the accuracy of UTCI forecasts and thus help with early warning of extreme weather events related to thermal stress.

## Supplementary Information

Below is the link to the electronic supplementary material.

Supplementary file 1 (pdf 1580 KB)
