# Supplementary material for: Improving the operational forecasts of outdoor Universal Thermal Climate Index with post-processing
Source: Int J Biometeorol. 2024 Mar 5;68(5):965–77. doi: 10.1007/s00484-024-02640-6 (PMC11636700; doi:10.1007/s00484-024-02640-6)
Supplement: Supplementary file 1 — (pdf 1580 KB) [file 484_2024_2640_MOESM1_ESM.pdf]

Supplementary Materials to: Improving the operational forecasts of outdoor Universal Thermal Climate Index with post-processing

by Danijela Kuzmanović, Jana Banko and Gregor Skok

S1 Supporting tables and figures

Table S1 The six different setups with different sets of input parameters used with LR and NN. The input parameters: UTCI forecasted from ALADIN (UTCI(Al)), the hour of the day (hour), forecasted air temperature (T), forecasted relative humidity (f), forecasted wind speed ($v10m$), the difference between model and actual station altitude ($Dheight$), forecasted total cloud cover (tcc), forecasted mean radiant temperature (T_{mrt}), actual station altitude (alt), altitude of the station in the model ($mheight$).

Setup	No. of inputs	Input parameters
1.	7	UTCI(Al), hour, T, f, $v10m$, T_{mrt}, $Dheight$
2.	8	UTCI(Al), hour, T, f, $v10m$, tcc, alt, $mheight$
3.	3	UTCI(Al), hour, T
4.	9	UTCI(Al), hour, T, f, $v10m$, T_{mrt}, $Dheight$, tcc
5.	8	UTCI(Al), hour, T, f, $v10m$, T_{mrt}, alt, $mheight$
6.	7	UTCI(Al), hour, T, f, $v10m$, T_{mrt}, tcc

Table S2 The daily average values of ME and MAE for the test set for the post-processing of combined data from all the stations. The results are provided with respect to the six different setups described in Table S1.

	Neural Network		Linear Regression		Uncorrected ALADIN forecast	
Setup	ME	MAE	ME	MAE	ME	MAE
1.	0.41 °C	3.36 °C	0.00 °C	3.81 °C	2.57 °C	5.02 °C
2.	0.36 °C	3.05 °C	0.00 °C	3.48 °C	2.57 °C	5.02 °C
3.	1.18 °C	3.77 °C	0.00 °C	4.15 °C	2.57 °C	5.02 °C
4.	0.33 °C	3.34 °C	0.00 °C	3.80 °C	2.57 °C	5.02 °C
5.	0.15 °C	3.03 °C	0.00 °C	3.47 °C	2.57 °C	5.02 °C
6.	1.03 °C	3.65 °C	0.00 °C	4.04 °C	2.57 °C	5.02 °C

Table S3 LR coefficients for the post-processing of combined data from all stations for each of the six setups described in Table S1

Setup	UTCI(Al)	hour	T	f	$v10m$	T_{mrt}	$Dheight$	tcc	alt	$mheight$
1.	0.7308	0.0093	0.2878	-0.0336	0.0359	-0.1737	-0.0864			
2.	0.3169	0.0113	0.4631	-0.0028	-0.1420			-0.0086	-0.2555	0.1514
3.	0.4540	0.0162	0.3941							
4.	0.7322	0.0091	0.2860	-0.0276	0.0439	-0.1714	-0.0867	-0.0065		
5.	0.6352	0.0101	0.3272	-0.0217	0.0220	-0.1327			-0.2546	0.1538
6.	0.8141	0.0075	0.2826	-0.0344	0.0863	-0.2133		-0.0040		

Table S4 The daily average values of ME and MAE with post-processing done separately on the data from each of the nine selected stations shown with bold font in Figure 1 from the main manuscript. Setup 5 was used for all the stations.

	Neural Network		Linear Regression		Uncorrected ALADIN forecast	
Setup	ME	MAE	ME	MAE	ME	MAE
Bilje	0.29 °C	2.25 °C	0.00 °C	2.61 °C	2.97 °C	3.94 °C
Celje	0.31 °C	2.23 °C	-0.02 °C	2.48 °C	1.61 °C	3.81 °C
Kredarica	0.08 °C	7.28 °C	-0.02 °C	7.44 °C	12.76 °C	14.55 °C
Airport JP Ljubljana	0.47 °C	2.24 °C	0.10 °C	2.45 °C	3.71 °C	4.32 °C
Ljubljana – Bežigrad	0.16 °C	1.86 °C	-0.03 °C	2.10 °C	1.38 °C	3.75 °C
Murska Sobota – Rakičan	-0.09 °C	2.29 °C	-0.01 °C	2.49 °C	0.56 °C	3.97 °C
Novo Mesto	0.18 °C	2.06 °C	-0.02 °C	2.37 °C	2.05 °C	3.63 °C
Portorož airport	0.17 °C	2.85 °C	-0.04 °C	3.33 °C	2.23 °C	4.69 °C
Rateče	-0.09 °C	1.98 °C	0.03 °C	2.30 °C	0.88 °C	3.72 °C

Table S5 LR coefficients for post-processing done separately on the data from each of the nine selected stations shown with bold font in Figure 1 from the main manuscript. Setup 5 was used for all the stations.

Station	UTCI (Al)	hour	T	f	$v10m$	T_{mrt}
Bilje	0.7413	0.0143	0.3011	-0.0113	0.0548	-0.2337
Celje-Medlog	0.4281	0.0119	0.5593	-0.0319	-0.0508	-0.0753
Kredarica	0.2416	0.0053	0.5737	-0.0306	-0.3431	-0.0014
Airport JP Ljubljana	0.4442	0.0155	0.4209	-0.0153	-0.0561	-0.0563
Ljubljana-Bežigrad	0.5041	0.0107	0.5508	-0.0253	-0.0321	-0.1430
Murska Sobota-Rakičan	0.3942	0.0140	0.5110	-0.0171	-0.0347	-0.0570
Novo Mesto	0.4304	0.0186	0.5243	-0.0181	-0.0333	-0.0748
Portorož airport	0.1475	0.0347	0.5848	0.0465	-0.0635	0.1122
Rateče	0.2138	0.0212	0.6884	-0.0107	0.0018	0.0323

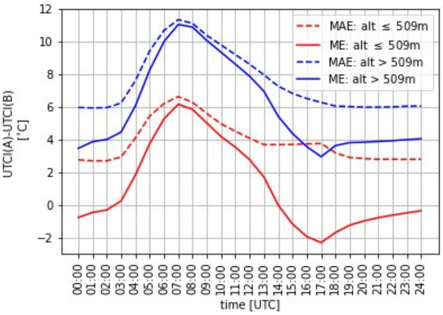

Fig. S1 Same as Figure 2a from the main manuscript, but with stations separated into two groups according to the altitude, with each group analyzed separately. The red color represents the group of stations with an altitude of less or equal to 509 m (there were 25 such stations), and the group with an altitude higher than 509 m by blue (there were 17 such stations).

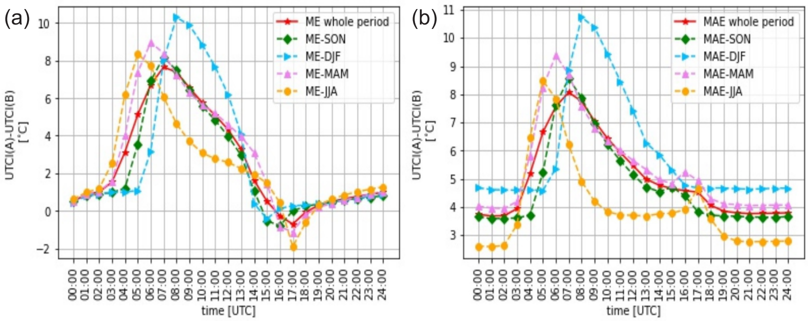

Fig. S2 Same as Figure 2a from the main manuscript but with analysis done separately for each season with (a) showing results for ME and (b) for MAE. SON (September, October, November) season is shown in green, DJF (December, January, February) in blue, MAM (March, April, May) in pink, JJA (June, July, August) in orange, and all seasons together in red color.

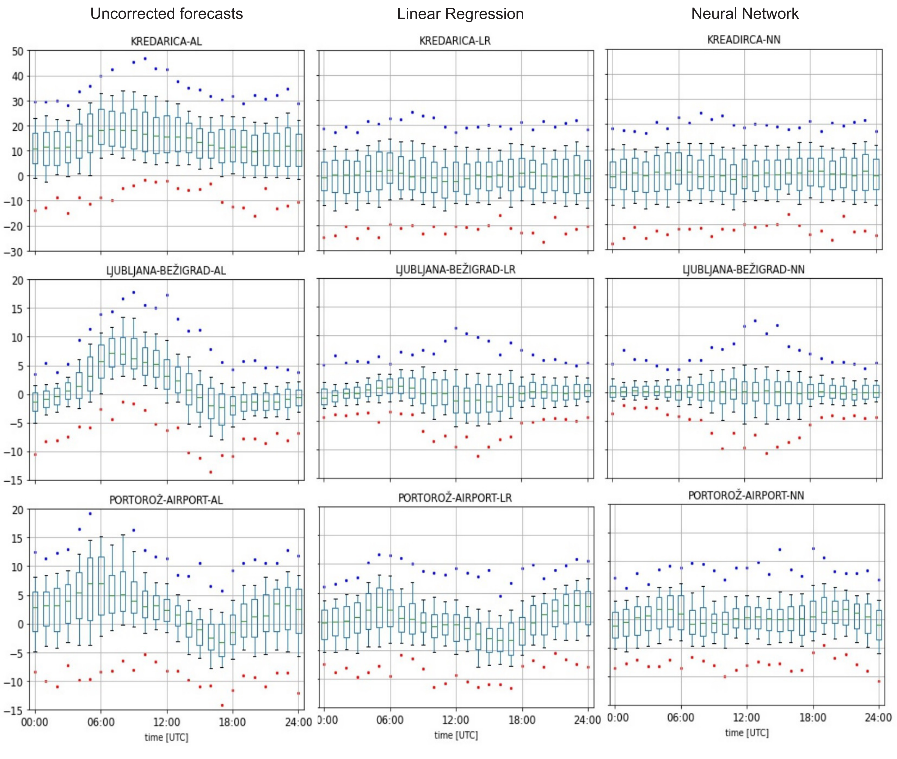

Fig. S3 Same as Figure 5 in the main manuscript, but for post-processing done separately on three individual stations: Kredarica, Ljubljana-Bežigrad, and Portorož Airport.

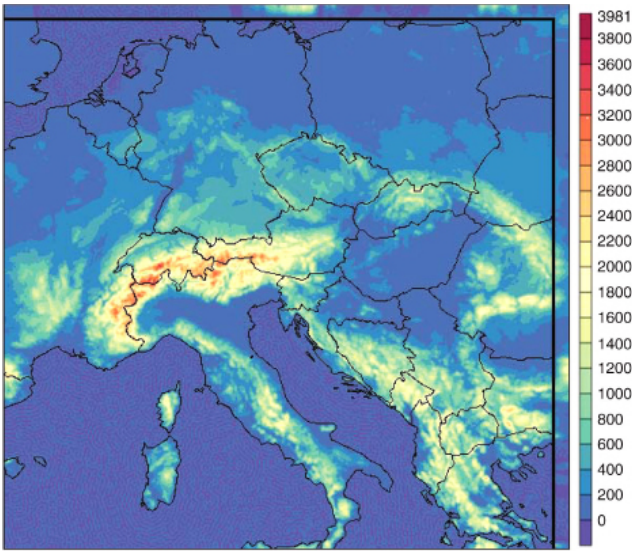

Fig. S4 The domain of the ALADIN model used operationally in Slovenia by the Slovenian Environment Agency. The colors denote the orography elevation in metres. The border of the extension zone is denoted with the black line. The figure was originally published under the CC BY 4.0 license in Blažica, V., Žagar, N., Strajnar, B. and Cedilnik, J., 2013. Rotational and divergent kinetic energy in the mesoscale model ALADIN. Tellus A: Dynamic Meteorology and Oceanography, 65(1), p.18918.DOI: https://doi.org/10.3402/tellusa.v65i0.18918.

Table S6 A list of meteorological stations in Slovenia that were used in the study. The nine stations that were analyzed individually are marked with bold font. The Altitude denotes the actual altitude of the station, while the Model Altitude denotes the altitude of the station in the orography used by the ALADIN model.

Name	Longitude [°]	Latitude [°]	Altitude [m]	Model altitude [m]
**BILJE**	**13.624**	**45.8956**	**55**	**80**
BORŠT PRI GORENJI VASI	14.1819	46.0854	564	591
BOVEC-LETALIŠČE	13.5538	46.3317	441	1006
**CELJE-MEDLOG**	**15.2259**	**46.2366**	**242**	**314**
CERKLJE-LETALIŠČE	15.5161	45.901	154	172
GAČNIK	15.6838	46.6178	292	289
GODNJE	13.8433	45.7547	320	352
ILIRSKA BISTRICA-KOSEZE	14.2355	45.5531	415	540
ISKRBA	14.858	45.5612	532	5873
KANIN	13.4744	46.3581	2260	1247
KOPER-KAPITANIJA	13.7246	45.5481	4	19
**KREDARICA**	**13.8489**	**46.3787**	**2513**	**1885**
KRVAVEC	14.5333	46.2973	1742	925
LENDAVA	16.4579	46.5526	159	193
LESCE-LETALIŠČE	14.1718	46.362	509	544
LETALIŠČE EDVARDA RUSJANA MARIBOR	15.6818	46.4797	264	246
**LETALIŠČE JOŽETA PUČNIKA LJUBLJANA**	**14.4784**	**46.2114**	**362**	**365**
LISCA	15.2849	46.0678	947	405
**LJUBLJANA-BEŽIGRAD**	**14.5124**	**46.0655**	**299**	**271**
MALKOVEC	15.2049	45.9531	397	333
MARIBOR-VRBANSKI PLATO	15.626	46.5678	279	346
**MURSKA SOBOTA-RAKIČAN**	**16.1913**	**46.6521**	**186**	**194**
NANOS	14.0536	45.7714	1241	733
**NOVO MESTO**	**15.1773**	**45.8018**	**220**	**248**
PODČETRTEK-ATOMSKE TOPLICE	15.6083	46.1547	202	239
**PORTOROŽ-LETALIŠČE**	**13.616**	**45.4753**	**2**	**70**
POSTOJNA	14.1973	45.7722	538	608
**RATEČE**	**13.7129**	**46.4971**	**864**	**987**
RAVNE NA KOROŠKEM	14.94	46.5477	396	671
ROGAŠKA SLATINA	15.6439	46.2409	289	303
ROGLA	15.3315	46.453	1495	1264
SLAVNIK	13.976	45.5336	1020	704
SOTINSKI BREG	16.0307	46.8359	415	319
TOPOL	14.3713	46.0941	692	466
TROJANE-LIMOVCE	14.9113	46.1984	673	550
VEDRIJAN	13.541	46.0131	232	177
VELIKE LAŠČE	14.6427	45.831	528	547
VOGEL	13.8396	46.2594	1515	1087
ZGORNJA KAPLA	15.3501	46.6434	722	625
ČRNOMELJ-DOBLIČE	15.1462	45.56	157	308
ŠEBRELJSKI VRH	13.9113	46.0629	1066	628
ŠMARTNO PRI SLOVENJ GRADCU	15.1112	46.4896	444	616

S2 Case study examples

We show two case study examples for two winter days when large errors in UTCI forecasts occurred. Figure S5 shows the daily evolution of various meteorological parameters related to UTCI forecasts for three stations from different parts of the country (Portorož-Airport, Ljubljana-Bežigrad and Kredarica).

The left panels in Figure S5 show the February 28, 2018 results, which was a cold, calm and clear winter day. In Portorož (Figure S5a), the wind blew with a maximum speed of 7 m/s during the night, after which it calmed down to 2 m/s. The minimum daily temperature was -5.6 °C, while the maximum was 1.2 °C. Overall, the daily evolution of wind and temperature was predicted quite accurately, while some differences are visible during the night when the model predicted a lower wind speed and a slightly lower temperature. A larger difference is visible in T_{mrt}, with the forecasted value being larger in the middle

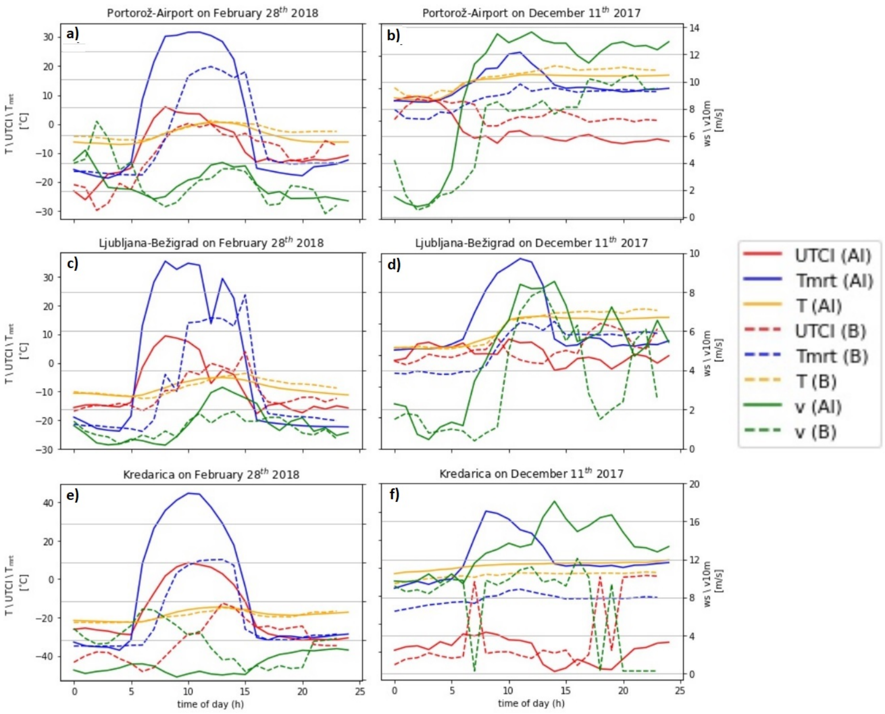

Fig. S5 Daily evolution of various meteorological parameters related to UTCI forecasts at three stations for two selected days. Forecasted values by the ALADIN (denoted with "(Al)") are presented by solid lines, while dashed lines (denoted with "(B)") represent the observed values (either measured or calculated from the measurements with the BioKlima software). The UTCI is plotted with red lines, the T_{mrt} with blue lines, air temperature with yellow lines and wind speed with green lines. The selected stations are Portorož-Airport (upper panels), Ljubljana-Bežigrad (middle panels) and Kredarica (bottom panels). The left panels represent February 28th, 2018, while the right panels represent December 11th, 2017. The left side of the y-axis represents the scales of T, T_{mrt} and $UTCI$, while the right side is for wind speed.

part of the day compared to the observed value. At midday, the difference was 10 °C, while in the morning, it was much larger (about 40 °C). There was a temporal shift between the T_{mrt} in the ALADIN model and observations, where ALADIN's forecasts were ahead by several hours. This might be caused due to errors in the forecasts of long-wave and short-wave fluxes. As a result, the forecasted UTCI values in the first half of the day were higher than the observed ones, while in the second half, the situation was reversed. Thermal stress changed during the day from slight cold stress to strong cold stress during the night, which is usual for that period of the year.

In Ljubljana (Figure S5c), the day was calm, with an average wind speed of about 1 m/s. The maximum daily temperature was -4 °C, while the minimum was -12.5 °C. The model forecast of wind and temperature was precise and accurate, while the UTCI forecast in the middle of the day differs by almost 20 °C and follows the course of the T_{mrt}. The difference in T_{mrt} values was likely caused due to errors in forecasting long-wave and short-wave fluxes. This can, for example, be caused by an error in the cloud cover forecast. The UTCI values were slightly higher than in Portorož, but thermal stress was in the same categories.

At Kredarica (Figure S5e), the day was also calm, with a light wind during the night. The maximum daily temperature was -14.8 °C, while the minimum was -23.3 °C. ALADIN forecasted a somewhat lower wind speed during the night, and the forecasted temperature followed the measured temperature throughout the day. The difference between the forecasted and measured values of T_{mrt} was the largest in the morning (around 40 °C), while it was 20 °C in the midday, which was comparable to the difference in UTCI values. It is likely that the difference between the station's altitude in the model and the actual altitude of the station (about 630 m - Table S6) may cause errors in cloudiness and radiation forecast, which can affect the errors in the T_{mrt} and UTCI forecasts.

The right side panels of Figure S5 show the results for December 11, 2017, which was a windy winter day. A wind of 10 m/s was blowing during the day in Portorož (Figure S5b). The minimum daily temperature was 7.5 °C, and the maximum was 16.9 °C. The model predicted a higher wind speed than was observed, with a difference of about 5 m/s. On the other hand, the forecasted temperature was lower than the measured. The mentioned errors led to a difference between forecasted and observed UTCI values of about 10 °C. Due to stronger wind and lower temperature, the model predicted lower UTCI values. Thermal stress during the day was slight cold stress.

In Ljubljana (Figure S5d), the wind was blowing with a maximum speed of 8 m/s. The measured temperature during the night was around 2 °C, while during the day, it has risen to a maximum of 15 °C. The model accurately predicted the daily evolution of temperature and wind, which led to a precise forecast of the UTCI course in the morning. During the evening, the model predicted a lower value of the UTCI than was observed, by roughly 15 °C, even though the forecasted T_{mrt} values were not much lower than the observed

ones. The error in UTCI forecast could be influenced by the errors in wind speed forecasts, which were lower than the observed for about 5 m/s. Thermal stress was slight cold stress.

At Kredarica (Figure S5f), the maximum wind speed was about 12 m/s while the daily temperature was around 0 °C. The model predicted a stronger wind in the second half of the day and a slightly higher temperature, with a difference of about 5 °C. Despite this, the model predicted mostly accurate UTCI values with only smaller errors. However, there are some unusual peaks in the observed UTCI values around 06:00 and 18:00, which align with the sudden drop in the measured wind speed to almost zero. Such sudden changes in wind speed can happen but could also be the result of measurement errors. As the forecasted wind speed does not exhibit such changes, the maximum difference between the forecasted and calculated UTCI value is large during these periods (approximately -30 °C).
